# Pitch-Based Activated Carbon Fibers: Activation Influences and Supercapacitor Applications

**DOI:** 10.3390/polym18020282

**Published:** 2026-01-20

**Authors:** Matthew Joe, Heon E. Park

**Affiliations:** Department of Chemical and Paper Engineering, Western Michigan University, 1903 W. Michigan Ave., Kalamazoo, MI 49008, USA; matthew.k.joe@wmich.edu

**Keywords:** pitch, polymers, activated carbon fibers, activated carbon nanofibers, pores, carbon fiber electrodes, supercapacitors, energy storage

## Abstract

Pitch-based activated carbon fibers, recognized for their excellent electrical conductivity, mechanical strength and durability, offer a compelling electrode alternative in the development of next-generation supercapacitors. In this review, we provide insight into the critical role of porosity in enhancing pitch-based carbon fiber performance in supercapacitors, with a focus on the processes and enhancements employed for pore introduction. The background and theoretical underpinnings for the necessity of porosity are briefly introduced, providing a rationale for the optimization of pore distribution. Moreover, the practical outcomes of these treatments are explored in supercapacitor applications, demonstrating the energy storage capabilities of pitch-based activated carbon fibers. In preparing this review, we surveyed the literature and found that pore introduction onto pitch-based carbon fibers is achieved almost solely through activation, which invites future research into alternative techniques. Additionally, it is apparent that future comparisons will benefit from the establishment of a standardized protocol for the reporting of supercapacitor performance.

## 1. Introduction

### 1.1. Background

Currently, fossil fuels still hold the highest share in energy generation, accounting for almost 60% of global generation [1]. However, as the reality of climate change sets in, the depletion of fossil fuels and rising global pollution are expected to drive the adoption of renewable energy generation. This shift is already apparent, with the contribution of renewables to global energy generation rising from approximately 1% to 13% between 2000 and 2021, primarily driven by wind and solar energy [1]. To meet the growing demand for renewable energy systems, significant advancements in energy storage devices (ESDs), such as batteries and supercapacitors (SCs), are essential, particularly to address the intermittent nature of renewable sources. The enhancement of energy and power densities to improve electrochemical performance and reduce costs, while increasing durability, is critical for effectively storing excess energy until it can be utilized.

Supercapacitors, also known as electric double-layer capacitors (EDLCs), are a promising next-generation technology offering incredible power density (>10,000 W/kg), an outstanding cycle life (>100,000 cycles) and superior durability while operating at close to 100% Coulombic efficiency [2,3,4]. They find applications in areas that require high and reliable power output, such as memory protection, military and aerospace applications, electric vehicles [5,6] and renewable energy storage. Additionally, hybrid battery–supercapacitor couplings exhibit promising symbiotic performance [7,8]. However, the economic viability and range of applicability of SCs are diminished by their dismal energy densities when compared to batteries [9].

Like the majority of ESDs, the performance of a supercapacitor is closely tied to the electrode and its properties. Supercapacitors, in particular, are heavily dependent on the surface area of their electrodes due to their reliance on the formation of an electric double layer for increased energy density [10,11,12,13]. Among others, carbon represents the most widely utilized electrode material. Its natural abundance, combined with its superior electrical conductivity and tunable structure and properties, makes it a popular choice for ESDs [3,8,14]. One-dimensional carbon fibers (CFs) are a particularly attractive form of carbon, known for their high mechanical strength, outstanding strength-to-weight ratio, thermal and chemical stability and excellent thermal and electrical conductivities [15,16,17,18,19,20,21]. Globally, polyacrylonitrile-based (PAN) carbon fibers make up 90–95% of total carbon fiber production, with pitch-based fibers accounting for most of the remainder. Despite PAN’s dominance, pitch-based carbon fibers have seen a resurgence, driven by growing demand for cost-competitive alternatives that offer comparable mechanical properties while exhibiting superior electrical conductivities [15,20,22]. However, pristine CFs often exhibit low to negligible specific surface areas (SSAs) [23,24,25,26,27,28], rendering them insufficient for use in SCs. Therefore, to optimize carbon fibers for use as supercapacitor electrodes, it is essential to enhance their specific surface area and porosity, which will subsequently improve their electrochemical properties.

In this review, we discuss the pore-introduction methods employed to produce pitch-based porous carbon fibers, in which activation plays a central role in generating activated carbon fibers. It is therefore pertinent to define the term ‘activation’. Classically, activation is defined as the process by which the latent adsorption capacity of an existing carbonaceous matrix is made accessible through chemical and/or thermal (physical) treatments, transforming it into a highly adsorptive material [29,30]. Since activation requires an existing carbonaceous matrix, the term ‘activated’ is not strictly applicable to porous carbon fibers in which (i) adsorption-active sites are engineered during synthesis rather than derived from a pre-existing matrix, and (ii) the matrix is not chemically or physically treated to unlock latent activity. For clarity, the broader term ‘porous carbon fibers’ will be used in this review to describe such materials.

In modern practice, the enhancement of adsorption capacity in carbon fibers is largely achieved through the development of porosity and accessible surface area. While the fabrication and modification of porous carbon fibers [21,31,32,33,34,35,36,37,38,39] and their applications in supercapacitors [2,3,4,40,41,42,43,44,45,46,47,48,49,50,51,52,53,54,55,56,57] have been reviewed by others, to the best of our knowledge, there is an absence of reviews focusing on the pore-forming techniques employed specifically for pitch-based carbon fibers that are intended for supercapacitor applications. In the interest of providing a broader overview of this topic, we discuss works on conventional pitch-based porous carbon fibers as well as works that deal with pitch-based porous carbon nanofibers. Herein, we provide background and an overview of methods that are employed to introduce porous structures onto pitch-based carbon fibers and nanofibers and progress in their applications as supercapacitor electrodes.

### 1.2. Electric Double-Layer Capacitors

**Figure 1:**
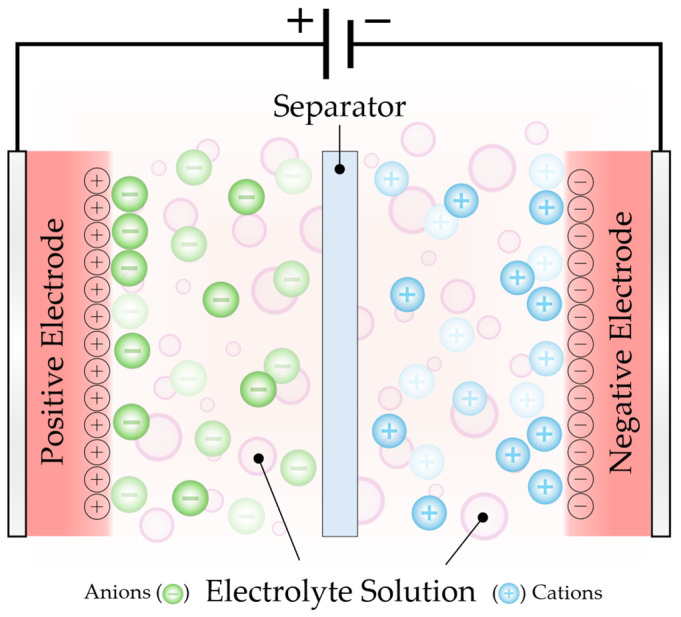
Electric double-layer capacitor schematic.

Much like batteries, supercapacitors consist of two electrodes in contact with an electrolyte that are isolated by a non-conducting separator (Figure 1) [8,58]. The key difference lies in their charge storage mechanisms. Batteries rely on charge transfer facilitated by redox reactions involving ion diffusion *across* the electrode–electrolyte interface into the bulk, where kinetics are governed by solid-state diffusion through the bulk electrode material [58,59]. On the other hand, supercapacitors store energy through physical electrostatic charge separation *at* the interface, which is a surface-controlled phenomenon [11,12]. When an external potential is applied across the electrodes, charged ions in the electrolyte accumulate at the electrode surface to compensate for the opposing charge of the electrode [60]. These ionic charges in the electrolyte undergo almost instantaneous physical adsorption/desorption, generating capacitance via electrostatic charge accumulation. The accumulation of charges on either side of the electrode–electrolyte interface constitutes what is known as the electric double layer [11,12,58,61,62].

### 1.3. Electric Double-Layer Theory

The performance of supercapacitors is fundamentally governed by the principles of electric double-layer (EDL) theory, which describes the behavior of ions at the interface between an electrode and an electrolyte. Over the past century or more, the development of EDL theory has been shaped by a series of key contributions, each addressing the limitations of earlier models and providing deeper insights into interfacial phenomena, which play a critical role in optimizing supercapacitor design and performance. The first formal model of the electric double layer was introduced by Helmholtz [11,13] in 1853, in which it was modeled as a rigid molecular capacitor, with a fixed layer of surface charges balanced by counterions in the electrolyte. While it assumed immobile ions, the model highlighted that electrodes with high surface area and porosity maximize capacitance by offering more sites for charge storage and energy accumulation [63,64]. In this model, the Helmholtz capacitance, *C_H_*, is described by the formula for a classical parallel-plate capacitor:

(1) $$C_{H}=\frac{\varepsilon_{r}\varepsilon_{0}A}{d_{H}}$$

where $\varepsilon_{r}$ is the relative permittivity of the electrolyte, $\varepsilon_{0}$ is the vacuum permittivity, *A* is the electrode surface area and *d_H_* is the thickness of the Helmholtz layer.

In the early 20th century, Gouy (1910) [65] and Chapman (1913) [66] addressed the limitations of Helmholtz’s model by proposing a diffuse double-layer model in which ions are distributed according to thermal motion, with concentration decaying exponentially away from the electrode. This more realistic model accounts for electrolyte concentration and temperature: where higher ion concentrations increase capacitance by packing more ions near the surface, though overcrowding limits this effect, while higher temperatures improve performance by reducing viscosity and enhancing ion mobility. At potentials close to zero charge, this diffuse-layer capacitance, *C_D_*, can be approximated by the following:

(2) $$C_{D}\approx \frac{\varepsilon_{r}\varepsilon_{0}A}{\lambda_{D}}$$

where $\lambda_{D}$ is the Debye length, defined as the characteristic distance from the charged electrode over which its electrostatic potential decays due to screening by the ions in the electrolyte.

Stern (1924) [12] reconciled the rigid and diffuse-layer models by introducing the concept of the Stern layer. It was proposed that the EDL consists of two regions: a compact inner layer (Stern layer), where ions are adsorbed due to both electrostatic and non-electrostatic forces, and a diffuse outer layer, where ion distribution is governed by the Gouy–Chapman model. This hybrid model offered a more complete EDL description, showing that ion adsorption at the electrode surface, especially in the Stern layer, contributes more to capacitance than ions in the diffuse layer. This hybrid approach allows the total double-layer capacitance, *C_EDL_*, to be expressed as a series combination of the Stern and diffuse layers:

(3) $$\frac{1}{C_{EDL}}=\frac{1}{C_{S}}+\frac{1}{C_{D}}$$

capturing contributions from both compact and diffuse ion distributions. Furthermore, Stern’s model emphasized the importance of pore size in electrodes, where pores must be large enough to allow ion access but small enough to maximize surface area [64].

Grahame (1947) [67] further refined Stern’s model by distinguishing between the inner Helmholtz plane (IHP) and the outer Helmholtz plane (OHP). Here, the IHP represents the location of specifically adsorbed ions that have lost their hydration shells, while the OHP marks the closest approach of ions that are still hydrated. Ions at the IHP contribute more to capacitance due to their proximity to the surface, highlighting the importance of electrolyte selection, as ions that can shed their hydration shells more easily (e.g., smaller ions or those with weaker hydration) tend to enhance capacitance [64].

**Figure 2:**
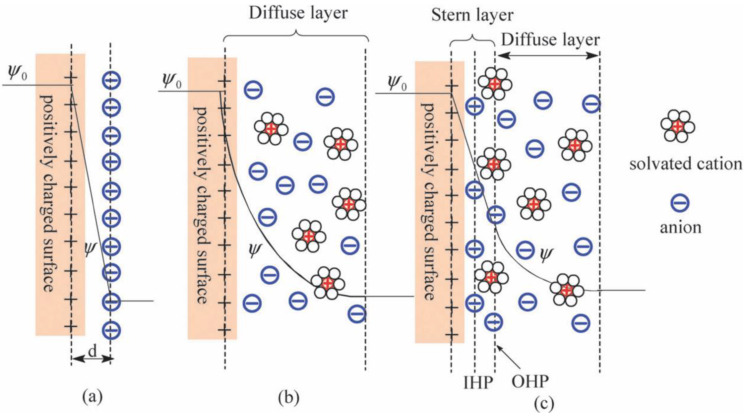
An illustration of the (**a**) Helmholtz model, (**b**) Gouy–Chapman model and (**c**) Stern model. *d* is the double-layer distance described by the Helmholtz model. *Ψ*_0_ and *Ψ* are the potentials at the electrode surface and the electrode–electrolyte interface, respectively. Reproduced with permission from [69], Copyright 2009, Royal Society of Chemistry.

The final major advancement in EDL theory came from Bockris, Devanathan and Müller (1963) [68], who incorporated the role of solvent effects and ion hydration into the model. They emphasized the importance of water molecules in structuring the double layer, particularly how the orientation of solvent molecules and the hydration of ions influence the overall behavior of the interface. This addition is critical for optimization as it explains how the choice of solvent and the hydration of ions impact energy storage. In aqueous electrolytes, the strong hydration of ions can limit their approach to the electrode surface, reducing capacitance. In contrast, non-aqueous electrolytes (e.g., organic or ionic liquids) often allow ions to come closer to the surface, increasing capacitance but at the cost of lower ionic conductivity [6,64]. Together, these contributions have established the modern framework of EDL theory (illustrated in Figure 2), which serves as the basis for designing higher performance supercapacitors. More importantly, it provides insight into why high-surface-area electrodes, hierarchical pore structures and tailored surface chemistries are essential for maximizing capacitance.

According to EDL theory, the capacitance of an electrode–electrolyte interface is primarily governed by the formation of an electric double layer, which scales with surface area. However, it is important to make the distinction between *available* surface area and *accessible* surface area; while the former may facilitate double-layer formation, the latter accommodates the favorable ion–electrode interactions that result in capacitance. In EDLC electrode design, it is important to maximize surface area while promoting fast ion transport by targeting hierarchical pore distributions [70]. As defined by the International Union of Pure and Applied Chemistry, pores can be classified according to their size, where *macropores* possess widths exceeding 50 nm; *mesopores* possess widths between 2 and 50 nm; and *micropores* possess widths not exceeding 2 nm [71]. Individually, each pore plays an important role in charge storage: while macropores do not contribute much surface area, they serve as ion reservoirs, ensuring electrolyte ions are always available near smaller pores; mesopores act as ionic highways, enabling fast ion transport to and from micropores; finally, micropores act as the primary charge storage sites [70].

In order to maximize the capacitance, ions must be subjected to a high degree of confinement to amplify the ion–electrode interactions within micropores; this requires the tailoring of pores to complement the electrolyte ion size [72,73]. As an ion is increasingly confined within a pore, the capacitance, *C*, increases in accordance with the following:

(4) $$C\propto \frac{\varepsilon_{r}\varepsilon_{r}A}{d}$$

where *A* is the surface area, *d* is the distance between the pore wall and center of the ion and $\varepsilon_{r}$ is the relative permittivity of the electrolyte. As *d* decreases, i.e., the size of the pore approaches the size of the ion, the capacitance increases; conversely, as the pore becomes larger, a decrease in capacitance is experienced. Thus, the minimization of free space within a pore will result in the greatest electric double-layer interaction between ion and electrode, where maximum capacitance is obtained when the pore size matches the ion size [74,75].

## 2. Carbon Fiber Fabrication

Amongst the allotropes of carbon, one-dimensional carbon fibers stand out as having high tensile strength, low density, high dimensional stability, high corrosion and thermal resistance and high electrical conductivity [19,20]. They are defined as having at least 92% carbon [76] and are typically derived from polyacrylonitrile, cellulose, or pitch. Among these precursors, this review focuses on pitch, which presents as a highly viscous, solid-like material at room temperature. After the refining of petroleum and coal tar, pitch remains as a complex, highly aromatic, heavy hydrocarbon residue known as petroleum and coal tar pitch, respectively [77]. It can possess more than 80% carbon content [15] and obtain a high degree of graphitization, making it a prime candidate for carbon fibers in energy storage applications. Regardless of precursor, carbon fiber production generally involves the spinning of the precursor into fibers, followed by a stabilization step and ending with carbonization [78].

### 2.1. Fiber Formation: Melt Spinning and Electrospinning

**Figure 3:**
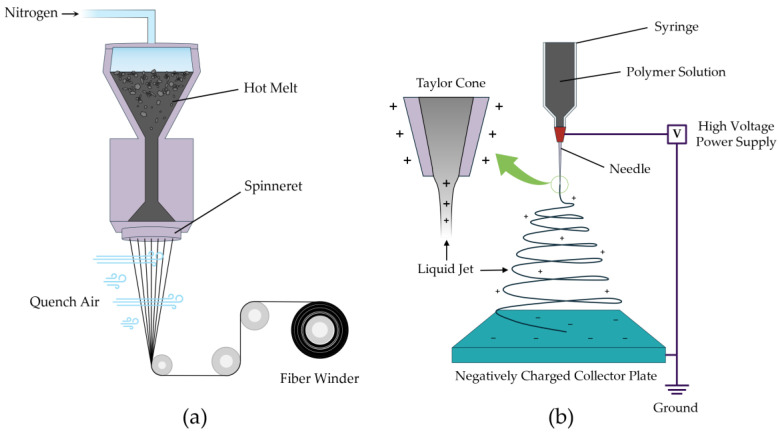
Schematics depicting (**a**) melt spinning and (**b**) electrospinning.

In order to form carbon fibers and carbon nanofibers, melt spinning and electrospinning are employed, respectively (illustrated in Figure 3). Melt spinning is a highly temperature-sensitive extrusion process where solid pitch is melted and extruded through a spinneret to form continuous filaments 12–30 µm in diameter. As the extruded filaments exit the spinneret, they cool and solidify into fiber form while being drawn and collected [20,79,80].

Electrospinning is a process that utilizes electrohydrodynamics to produce ultrathin fibers from a polymer solution. This process involves the application of high voltage to a pitch-based solution held in a syringe, which creates an electrically charged droplet at the tip of the needle. As the electrostatic forces overcome the surface tension of the droplet, a fine jet of liquid is ejected from the apex of the conical meniscus, known as a Taylor cone. This charged jet undergoes rapid stretching and thinning due to electrostatic repulsion and interactions with the external electric field. As the jet travels towards a grounded collector, the solvent evaporates or the material solidifies, resulting in the deposition of continuous fibers with diameters ranging from nanometers to micrometers [81,82].

### 2.2. Stabilization and Carbonization

To ensure that the structural integrity of fibers is maintained during carbonization, they must undergo oxidative stabilization to render the fibers infusible. This step is performed via thermal treatment in an oxidative atmosphere, where fibers are typically heated in air up to 230–350 °C. During this process, oxygen is introduced into the fiber structure through oxidation, dehydration, cross-linking and cyclization reactions [15], thereby thermosetting the fibers. Finally, carbonization is performed to obtain carbon fibers, where stabilized carbon fibers are heated to final temperatures between 900 and 1800 °C in an inert atmosphere. During this process, most of the non-carbon atoms are liberated from the carbon matrix through condensation and aromatization [15,20], consolidating the carbon structure.

## 3. Activated Carbon Fiber Fabrication

Naturally, if exceptional supercapacitor performance is desired, carbon fibers must possess exceptional surface area, complemented by favorable pore distributions to achieve a surface tailored for rapid ion transport and efficient adsorption. For pitch-based porous carbon fibers, activation is the prevailing, almost sole, method for pore introduction, yielding activated carbon fibers (ACFs), although simple thermal treatment and pyrolysis [83,84] can be sufficient. Thus, physical and chemical activation will be primarily discussed as well as enhancement methods [85,86,87,88] and potential alternative pathways to fabricate these pitch-based ACFs. The properties and activation conditions of various activated carbon fibers are summarized in Table 1.

### 3.1. Carbon Fiber Activation

#### 3.1.1. Physical Activation

Generally, physical activation is a solid–gas reaction in which a gaseous oxidizing agent strips carbon atoms from the surface of a precursor’s carbon matrix [121]. The voids left behind by the continual gasification of carbon atoms result in the formation of pores, which contribute to increasing surface area. A wide pore distribution is characteristic of physical activation and may be exacerbated as pores continue to widen upon prolonged activation. Due to the inherent nature of physical activation, whereby carbon atoms are plucked from the carbon matrix, the underlying fiber will often experience substantial surface damage, for which this burn-off (of these carbon atoms) is taken as the degree of activation. Consequently, the carbon yield of activated carbon fibers and activated carbon nanofibers (ACNFs) tends to suffer greatly over the course of activation [122]. For pitch-based materials, this process is typically carried out with CO_2_ or steam at temperatures ranging from 700 to 1000 °C, with the development of porosity predominantly being a function of activation time, temperature and agent, as well as the carbon structure of the underlying fiber—additionally, finer control can be achieved by utilizing carrier gases, such as nitrogen, to inhibit auxiliary reactions.

Being largely endothermic, these reactions require higher temperatures for favorable reactions to occur and proceed according to the following fundamental reactions [123]:

(5) $${CO}_{2}\left(g\right)+C\left(s\right)⇌2CO(g)\;\Delta H=162\;kJ/mol$$

(6) $$H_{2}O(g)+C(s)⇌CO(g)+H_{2}(g)\;\Delta H=118\;kJ/mol$$

The endothermic nature of these reactions affords a great deal of control when tuning the reaction rate and therefore the activation rate. From the enthalpic values, it is obvious that CO_2_ requires a higher heat input for the reaction to proceed compared to steam. As a result, steam will generally exhibit a higher reaction rate than CO_2_ at the same temperature [123]. Activation with steam tends to occur at the surface, resulting in a wider porous texture with a pronounced decrease in fiber diameter; while activation with CO_2_ tends to take place within the fiber, due to its larger coefficient of diffusion. As a result, CO_2_ is able to develop porosity deep within the fiber, with a negligible decrease in fiber diameter. Consequently, CO_2_ is able to deliver a higher proportion of narrow micropores compared to steam. However, activation deep within the fiber results in a decrease in tensile strength [124], while a higher degree of burn-off hinders the electrical conductivity of the fibers [125].

#### 3.1.2. Chemical Activation

Conversely, chemical activation is a method that is able to achieve higher carbon yields and a relatively narrow pore size distribution, with lower temperatures in the range of 300–900 °C [123], with activation performance being predominantly a function of reagent concentration, time and temperature. It is generally considered a solid–solid reaction, in which the underlying carbon material is mixed and impregnated with the chemical reagent and then heated in an inert atmosphere to generate porosity. Typical reagents include strong dehydrating species such as zinc chloride (ZnCl_2_), phosphoric acid (H_3_PO_4_) and metal hydroxides such as potassium hydroxide (KOH) and sodium hydroxide (NaOH) [123,126]. While dehydrating agents are typically used for activating biomass, due to their high hydrogen and oxygen content, metal hydroxides are favored for carbon-rich precursors, as their graphitic structures allow for intercalation, in addition to etching of the surface [126].

Though corrosive, KOH is the most common reagent for pitch-based carbons and follows the fundamental reaction below [123]:

(7) $$6KOH+2C\to 2K+3H_{2}+2K_{2}CO_{3}$$

In addition to multiple simultaneous and consecutive reactions, KOH reacts with the carbon to form metallic potassium and its carbonate. Upon heating, products of the reaction may decompose and etch the surface of the carbon fibers, leaving behind pores and intermediates that further attack the surface. A distinctive and highly effective property of these metallic hydroxides is the ability of the reduced metallic ions to enter the carbon matrix by way of intercalation. As these ions enter between the graphene sheets, they create pockets within the carbon matrix, introducing porosity. In order to realize this porosity, the intercalated metals must be released from within the carbon matrix. This is usually achieved via a post-activation washing process in which acidic or basic solutions are used to remove reaction residues and intercalated metals [126]. If higher SSA and pore volumes are desired, the KOH-to-carbon ratio can be increased. However, the microporosity and yield will eventually suffer [106,107].

### 3.2. Determinants of Activation

#### 3.2.1. Effect of Fiber Diameter on Activation Rate

**Figure 4:**
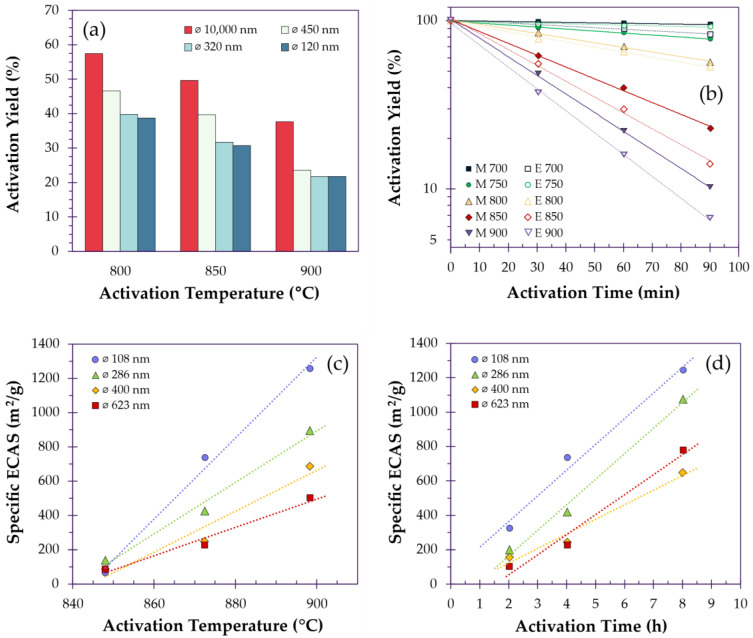
(**a**) CNFs of different diameters activated at various temperatures, redrawn based on data reported by Tavanai et al. [127]. (**b**) Comparison between the activation of melt-spun (M) and electrospun (E) carbon fibers at various temperatures, redrawn based on data reported by Park et al. [91]. (**c**) Electrochemically accessible surface area (ECAS) generated for fibers of varying diameters across increasing temperatures (activated for 4 h). (**d**) Time scales (activated at 873 °C), redrawn based on data reported by Erben et al. [125]. NOTE: Trendlines present in this figure are intended only for visual guidance.

To discuss both ACFs and ACNFs, it is important to understand how their inherent characteristics may manifest during the activation process. The most obvious distinction is the difference in fiber diameter, where the specific surface area (SSA) is fundamentally larger for CNFs. During activation, this inherent difference is reflected as an increase in the activation rate. For instance, Tavanai et al. prepared CNFs of varying diameters ranging from 120 to 450 nm for activation with CO_2_ at different temperatures (800–900 °C). ACNFs with finer diameters consistently obtained higher SSAs and experienced higher burn-off regardless of activation temperature, for the same activation time (Figure 4a). Activation was also performed on a carbon fiber with a diameter of 10 µm, in which the smallest ACNF was able to obtain an SSA of over 9× the larger fiber [127]. Park et al. compared the performance of melt-spun fibers versus electrospun pitch-based fibers when activated with steam between 700 and 900 °C, with diameters of 10 µm and 1 µm, respectively (Figure 4b). To quantify the performance of the fibers of different diameters, they determined the rate constant of activation, for both fiber types, to be 13–20 times larger for the electrospun fibers, owing to the finer diameter, in which the non-activated electrospun fibers initially possessed surface areas 6.4 times greater than the melt-spun fibers [91]. However, it should be noted that the SSA of electrospun fibers is often diminished due to fiber fusion [91,128], but finer fibers still retain higher activation kinetics, particularly for physically activated fibers, as it takes reagents longer to penetrate and diffuse through thicker fibers (Figure 4c,d) [125].

#### 3.2.2. Effect of Crystallinity on Activation Efficiency

When comparing ACFs and ACNFs, crystallinity must be acknowledged, as melt-spun pitch-based carbon fibers are typically able to achieve higher degrees of alignment, possessing highly ordered crystalline structures, compared to electrospun fibers. Typically, carbons located at the edges of graphene sheets, defect positions and discontinuities are more reactive, and as a structure becomes more ordered, carbons in these positions become rarer, making it increasingly difficult for carbons to be stripped from the matrix [121,123]. This effect is more pronounced in CNFs, particularly when physically activated, as the disorder enables activation agents to penetrate further into the carbon matrix, intensifying the activation effect [91,93]. On the other hand, when chemically activated, for example, with KOH, highly crystalline structures may prevent metallic ions from intercalating into the structure, resulting in lower surface areas [116,129].

**Figure 5:**
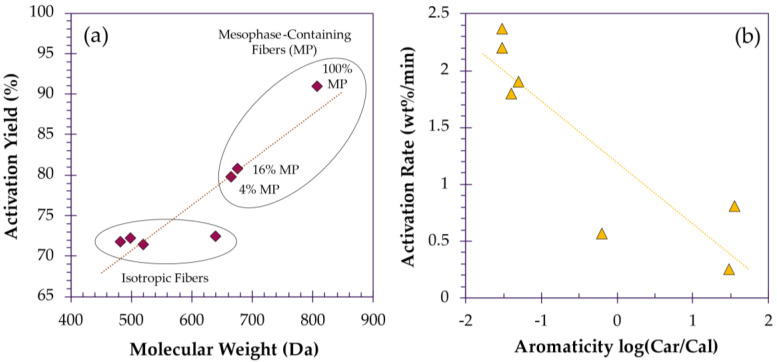
(**a**) Effect of fiber anisotropy on activation efficacy, redrawn based on data reported by Tekinalp et al. [102]. (**b**) Activation rate as a function of pitch aromaticity, redrawn based on data reported by Derbyshire et al. [25]. NOTE: Trendlines present in this figure are intended only for visual guidance.

Interestingly, when the molecular weight of the precursor pitch is increased, the degree of activation is affected minimally until crystallinity is introduced. Tekinalp et al. demonstrated this by preparing ACFs from seven fractions of petroleum pitch with increasing molecular weight (MW) and anisotropy for activation in CO_2_ at 840 °C for 6 h. During activation, the weight loss of isotropic fibers was independent of MW. However, in the presence of molecular order, the activation process is significantly slowed, resulting in a dramatic decrease in pore formation (Figure 5a). Not only were pores fewer, but they were larger. Isotropic fibers possessed a significant proportion of smaller pores (3.9–6.8 Å), while anisotropic fibers possessed fewer smaller pores, but a greater proportion of larger pores (>13 Å), which can be attributed to the presence of a highly crystalline mesophase. The higher order pitches were less susceptible to activation due to the scarcity of accessible defect positions. The rarity of these higher reactivity carbons means that activation is likely concentrated there, as it is the point of least resistance, resulting in pore expansion, rather than pore formation [102]. This effect is also observed when comparing more aliphatic petroleum pitches with more aromatic coal tar pitches (Figure 5b) [25,26]. Conversely, it is also possible that the activation process disrupts the crystalline structure of the target fibers. Wang et al. prepared pitch-based ACFs, observing that, with activation and increasing activation temperature (650–950 °C), the diffraction peak intensity of the (002) crystal plane disappears, indicating that the graphitic structure of the fibers becomes increasingly disordered. This was attributed to the deep etching achieved by KOH in which intercalation of the metallic potassium can also play a role in disruption of the crystalline structure, resulting in highly microporous fibers [104,130].

#### 3.2.3. Effect of Temperature on Surface Area and Pore Development

**Figure 6:**
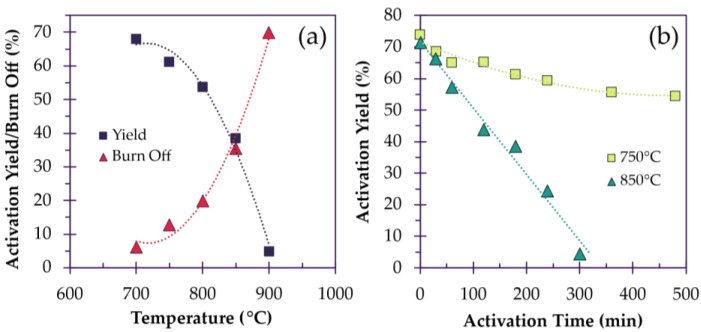
(**a**) The effect of increasing activation temperature on the carbon yield (activated for 180 min) and (**b**) a comparison between the effect of time and temperature on the carbon yield, redrawn based on data reported by Yue et al. [92]. NOTE: Trendlines present in this figure are intended only for visual guidance.

As micropores bring the most value in terms of surface area by volume, it is important to prioritize their formation to a certain extent, being mindful that a hierarchical pore distribution is necessary for the best electrode performance. During activation, the fiber properties can be affected largely by activation temperature and, to the lesser extent, by activation time (Figure 6a,b). For instance, Kim et al. prepared pitch-based ACNFs that were subjected to steam activation at 600 °C, 700 °C and 800 °C. As the temperature increased, ACNFs exhibited an increasing BET SSA of 590 m^2^/g, 774 m^2^/g and 939 m^2^/g. However, microporosity (that is, micropore volume by total pore volume) decreased from 81% to 76% to 55%, respectively [98]. These results are consistent with Bui et al., in which fibers were activated with steam at 700–900 °C. As the activation temperature was increased, the micropore ratio decreased from 79.5% to 49.2%, respectively, while the mesopore ratio increased inversely, with final BET SSAs increasing from 722 m^2^/g, 1220 m^2^/g and 1877 m^2^/g [94]. Clearly, as the temperature is increased, mesopore formation is favored. Generally, at activation temperatures past 800–850 °C, mesoporosity starts to dominate. While not completely undesired, mesopores inherently have a lower surface area, which can affect the total surface area of the ACFs. However, this can become an issue when mesopores are generated at the expense of micropores via enlargement. This effect is apparent in ACFs produced by Yue et al. where fibers were activated at 800 °C, 850 °C and 900 °C for 3 h each. Microporosity generated at 800 °C and 850 °C increased from 78% to 83%, whereas prolonged activation at 850 °C resulted in diminishing returns with microporosity reaching 86%, in addition to the BET SSA stagnating at ~1980 m^2^/g after 4–5 h. However, an increase to 900 °C causes a dramatic decrease in microporosity, which drops to 64%, with a decrease in micropore volume but an increase in mesopore volume; implying some mesoporosity was created at the expense of micropores [92]. In contrast, Wang et al. activated fibers with KOH between 650 and 950 °C, with the micropore ratio decreasing from 75.9% to 57.2%. However, in this case, the total micropore surface area stagnates at ~1770 cm^2^/g with increasing SSA, suggesting that micropores are not being cannibalized to form mesopores, allowing for steady SSA increases due to the retention of micropores [104].

#### 3.2.4. Effect of Time on Porosity and Yield

**Figure 7:**
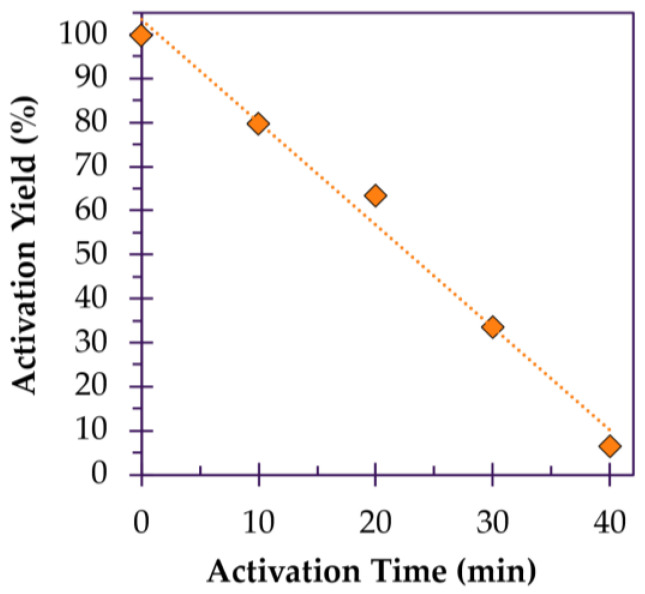
Carbon yield of activated carbon fibers over prolonged activation, redrawn based on data reported by Lee et al. [89]. NOTE: Trendlines present in this figure are intended only for visual guidance.

The effect of activation time is less subtle in its manifestation. Typically, as activation is prolonged, time manifests as a dismal return in carbon yield and excessive pore development. This applies for physical activation in particular, as fibers are continuously and indiscriminately stripped of carbon atoms, and less so for chemical activation [131]. For example, Lee et al. activated pitch-based fibers with steam at 900 °C for 10, 20, 30 and 40 min. After 10, 20 and 30 min, carbon yields of ~80%, 64% and 34% are obtained, respectively. Although an impressive BET surface area of 3230 m^2^/g is achieved, the carbon yield is just 6.5% (Figure 7), while microporosity has decreased, overdeveloping into mesopores [89].

#### 3.2.5. Effect of Modifiers on Pore Development

Should more mesoporous and macroporous [95] fibers be desired, there are certain methods that may result in a targeted pore distribution, including doping to enhance pore formation. For instance, Song et al. prepared pitch-based CNFs that were doped with nickel for activation in CO_2_, where the nanoparticles were observed to catalyze the characteristic pore expansion from physical activation. The nickel nanoparticles were particularly effective in catalyzing the expansion of micropores by facilitating the preferential gasification of carbon atoms from micropore walls, resulting in their expansion to meso- and macropores. Consequently, doped ACNFs experienced decreases in microporosity when nickel content was increased, with BET isotherms confirming the presence of a hierarchically porous structure [101]. Ryu et al. found the same effect with the addition of silver particles [132]. However, excessive doping led to pore blockage and fiber fracture [101]. Tamai et al. sought to produce mesoporous ACFs by experimenting with the addition of organometallic complexes to pitch-based carbon fibers. Yttrium acetylacetonate [Y(acac)_3_] was found to promote the formation of mesopores, resulting in ACFs exhibiting up to 80% mesoporosity. Compared to undoped ACFs, certain Y(acac)_3_-doped ACFs were able to achieve comparable SSAs, but carbon yields suffered with the enhanced activation rates [133,134,135]. Similarly, Lee et al. experimented with the addition of silver particles to pitch fibers for activation with CO_2_. Interestingly, during activation, silver particles were observed to bore and tunnel through the fiber, leaving channels behind that contributed to the meso- and macroporosity [136].

## 4. Alternative Pore Introduction Methods

As previously mentioned, activation is the predominant method for introducing porosity in pitch-based carbon fibers. Alternative approaches have been explored. However, their application to pitch-based carbon fibers is far less frequently reported in the literature. Nevertheless, we briefly summarize the few studies that have employed these alternative strategies and explore potential avenues.

### 4.1. Phase Separation

In the absence of an activation step, melt-spun carbon fibers are less likely to develop significant porosity independently due to their relatively low volatile content and the dense, solvent-free nature of the melt. On the other hand, the inherent use of a polymer–solvent solution for electrospinning often results in fibers with intrinsic porosity [83,117,118,119]. By controlling the solidification of the ejected fiber jet during electrospinning, phase separation between the polymer and solvent occurs, generating porous structures on the surface and throughout the bulk of each nanofiber. This phase separation can be induced by spinning fibers directly into cryogenic liquid, altering the relative humidity or tuning the contents of the polymer–solvent solution. Once phase separation is induced, polymer-rich and polymer-poor domains form, with polymer-rich regions solidifying into the nanofiber matrix, while the polymer-poor regions transform into porous structures upon solvent evaporation [82]. Additionally, when electrospun nanofibers are collected at sufficiently high areal densities, tight packing and extensive fiber–fiber entanglements occur, leading to the formation of a self-supporting fibrous network. Although individual fibers may exhibit micro- or mesoporosity, the random stacking and incomplete packing of the entangled fibers generate interconnected macropores within the bulk mat. These macropores originate from the interstitial voids between adjacent fibers and fiber bundles and are governed primarily by fiber diameter, orientation and collection density rather than by the intrinsic porosity of individual fibers. As a result, the electrospun mat exhibits hierarchical porosity, comprising intra-fiber pores and inter-fiber macroporous channels [137]. Subsequent stabilization and carbonization steps preserve these porous structures in the resulting carbon fibers [138]. For instance, without an activation step, Tian et al. produced pitch-based CNFs with BET SSA ranging from 437 to 643 m^2^/g with a hierarchical pore structure [139]. Similarly, Qi et al. obtained CNFs of BET SSA 365–543 m^2^/g with micro- and mesoporosity, demonstrating that considerable surface area can be intrinsically available [139,140].

### 4.2. Sacrificial Templating

Templating strategies have been widely employed as a means of deliberately generating and controlling porosity in carbon materials derived from a range of precursors [141,142,143,144]. These approaches are generally categorized into soft and hard templating methods based on the nature of the pore-directing agent. Soft templates typically involve molecular or supramolecular assemblies, such as surfactants or block copolymers [145,146], which self-assemble into ordered domains that act as transient pore formers; removal of these species through thermal decomposition during carbonization results in the formation of ordered pore networks. In contrast, hard templating relies on rigid, pre-formed scaffolds—such as silica [147,148], metal oxides [149] and molten salts [150,151]—that physically define pore geometry by constraining carbon deposition; subsequent chemical or thermal removal of the template yields carbons with well-replicated and narrowly distributed pore structures. To the best of our knowledge, only a handful of studies have employed templating as a method to alter the structure of pitch-based carbon fibers. For example, Yang et al. utilized silica templates to physically shape and conform molten pitch, resulting in carbon nanofiber bundles. However, surface area and porosity were negligible, as expected [152]. In contrast, Chan et al. utilized alumina templates with mesophase pitch to produce nanofibers with orthogonally arranged graphene layers relative to the fiber axis, resulting in fibers with “platelet symmetry”. Though not discussed in context, the arrangement of these graphene layers allows for the exposure of edge and defect sites that can contribute to the surface area [153]. Furthermore, pitch was solubilized in solvent and processed into hollow carbon nanofibers, after which solvent evaporation produced a unique cellular structure. Described as one-dimensional cellular foams, these nanofibers contain vesicles and cavities that likely elevate the surface area, though this was not explored in the paper [153]. Although templating approaches have not been extensively reported for pitch-based fibrous forms of carbon, several studies have demonstrated the effective generation of pores in different forms of carbon derived from pitch [141,154,155,156,157,158,159,160,161,162,163,164], highlighting the potential to extend these methods to pitch-based carbon fibers.

## 5. Activated Carbon Fibers as Electrodes in Supercapacitors

Tuning the surface morphologies and properties of ACFs to optimize electrode–electrolyte interaction is imperative in achieving high supercapacitor performance and arguably the most important aspect of electrode design. Pores that are tailored to the size of solvated electrolyte ions increase capacitance and decrease diffusive resistance. Functionalization enhances surface wettability and promotes pseudocapacitance, while the incorporation of hierarchical pore size distributions enhances ion transport, affording greater rate capability [108]. The supercapacitor performance of various ACF electrodes is summarized in Table 2.

### 5.1. The Role of Hierarchical Pore Constituents

#### 5.1.1. Effect of Surface Area and Microporosity on Capacitance

**Figure 8:**
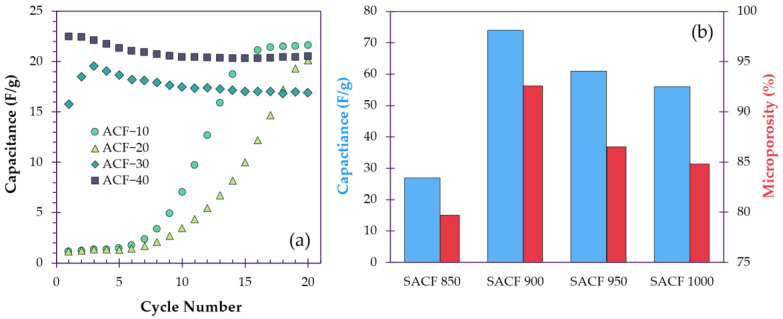
(**a**) Capacitance recovery with sufficient micropore wetting, redrawn based on data reported by Lee et al. [89]. (**b**) Capacitance achieved by ACFs activated at various temperatures, accounting for microporosity, redrawn based on data reported by Cho et al. [99].

As mentioned previously, while high SSA is desirable, if the effective surface area is not accessible or conducive to charge storage, the extra surface area becomes redundant when targeting high capacitance. Thus, for maximal charge storage, micropores become indispensable. For instance, Lee et al. prepared ACFs possessing BET SSAs of 1520 m^2^/g and 3230 m^2^/g, with microporosities of 95% and 44%, exhibiting capacitances of 21.6 F/g and 20.6 F/g at 2 mA/cm^2^ in 1 M (C_2_H_5_)_4_NBF_4_, respectively. Despite the impressive SSA, ACFs with less than half this SSA were able to eclipse the latter in capacitance, owing to high microporosity, demonstrating that sheer surface area alone cannot trump the charge storage capabilities of micropores. However, it is worth noting that the highly microporous ACFs did require sufficient wetting before they were able to operate at their full potential (Figure 8a) [89]. Similarly, Cho et al. produced ACFs of increasing surface area (584, 718, 752 and 920 m^2^/g), complemented by increasing microporosity (80%, 85%, 87% and 93%), which corresponded to an increasing capacitance (26, 56, 61, and 74 F/g at 1 A/g in 6 M KOH) [99] (Figure 8b).

**Table 2:** Supercapacitor performance of various activated carbon fiber electrodes.

Electrode Material	BET Surface Area (m^2^/g)	Microporosity (%)	Test Cell Setup	Electrolyte ^‡^	Capacitance (F/g)	Current Density	Capacitance Retention	Cycle Retention	Ref.
Low SP Pitch/Polystyrene ACNFs	2169	–	Half-cell	0.8 M KPF_6_ in EC/DMC	61.9	0.05 A/g	50% from 0.05 A/g to 5 A/g	83.5% after 10,000 cycles at 1 A/g	[109]
Mesophase Pitch ACFs	1222	83.67%	Symmetrical Two-Electrode	6 M KOH	427	0.1 A/g	–	99.1% after 1000 cycles at 0.1 A/g	[112]
Pitch-based Asphaltene ACFs	2290	69.00%	Symmetrical Two-Electrode	6 M KOH	311	40 mA/g	71% from 40 mA/g to 4 A/g	91% after 10,000 cycles at 1 A/g	[107]
Boron-Manganese-doped Pitch/PAN ACNFs	718	–	Symmetrical Two-Electrode	6 M KOH	208	1 mA/cm^2^	–	90% after 3000 cycles at 1 mA/cm^2^	[117]
Manganese-doped Pitch/PAN ACNFs	620	–	Symmetrical Two-Electrode	6 M KOH	188	1 mA/cm^2^	83% from 1 mA/cm^2^ to 20 mA/cm^2^	95.8% after 3000 cycles at 1 mA/cm^2^	[119]
Boron-doped Isotropic Petroleum Pitch/PAN ACNFs	641	43.00%	Symmetrical Two-Electrode	6 M KOH	180	1 mA/cm^2^	92% from 1 mA/cm^2^ to 20 mA/cm^2^	–	[118]
Anthracene Oil-based Isotropic Pitch ACFs	891	93.00%	Symmetrical Two-Electrode	6 M KOH	146	1 A/g	50% from 1 A/g to 20 A/g	–	[100]
Pitch/PAN ACNFs	1724	49.20%	Symmetrical Two-Electrode	6 M KOH	143.5	1 mA/cm^2^	–	–	[96]
Pitch/PAN ACNFs	966	–	Symmetrical Two-Electrode	6 M KOH	130.7	1 mA/cm^2^	50% from 1 mA/cm^2^ to 20 mA/cm^2^	–	[83]
Pitch ACFs	2460	63.40%	Symmetrical Two-Electrode	1.0 M H_2_SO_4_	109	22.1 mA/cm^2^	–	–	[105]
Mesophase Pitch ACFs	2353	–	Symmetrical Two-Electrode	1 M Et_4_NBF_4_/PC	54	10 mA/cm^2^	–	97% after 20 cycles	[106]
Pitch ACFs	3230	43.90%	Symmetrical Two-Electrode	1 M Et_4_NBF_4_/PC	22.5	2 mA/cm^2^	–	91.4% after 20 Cycles	[89]
Pitch/PAN ACNFs	643	–	Asymmetrical Three-Electrode	6 M KOH	187	1 A/g	86% from 1 A/g to 100 A/g	–	[139]
Pitch/PAN ACNFs	543	75.86%	Asymmetrical Three-Electrode	6 M KOH	197	0.2 A/g	73% from 0.2 A/g to 1 A/g	–	[140]
Pitch ACFs	987	84.30%	Three-Electrode	1 M H_2_SO_4_	119	5 mV/s	93% from 5 mV/s to 50 mV/s	–	[114]
Pitch ACFs	920	92.60%	Three-Electrode	6 M KOH	74	1 A/g	–	–	[99]
Pitch-based Asphaltene ACFs	2233	84.94%	Three-Electrode	1 M H_2_SO_4_	482	1 A/g	66% from 1 A/g to 50 A/g	–	[108]
			Symmetrical Two-Electrode	1 M H_2_SO_4_	239	1 A/g	–	94.3% after 10,000 cycles at 1 A/g	
Low SP Pitch ACFs	1504	75.71%	Three-Electrode	6 M KOH	334	0.5 A/g	73.7% from 0.5 A/g to 50 A/g	91.4% after 10,000 cycles at 1 A/g	[111]
			Symmetrical Two-Electrode		230	0.5 A/g	–	–	
Coal Tar Pitch ACNFs	550	–	Three-Electrode	6 M KOH	235	0.5 A/g	50% from 1 A/g to 20 A/g	–	[115]
			Symmetrical Two-Electrode		46.7	0.1 A/g	–	99.2% after 10,000 cycles at 10 A/g	
Isotropic Pitch ACFs	286	98.00%	Three-Electrode	6 M KOH	150	0.1 A/g	60.7% from 0.1 A/g to 10 A/g	89.1% after 3000 cycles at 5 A/g	[116]
			Symmetrical Two-Electrode		86		–	–	

^‡^ KPF_6_ in EC/DMC = potassium hexafluorophosphate in ethylene carbonate/dimethyl carbonate, KOH = potassium hydroxide, H_2_SO_4_ = sulfuric acid, Et_4_NBF_4_/PC = tetraethylammonium tetrafluoroborate in propylene carbonate.

#### 5.1.2. Effect of Mesoporosity on Rate Performance

**Figure 9:**
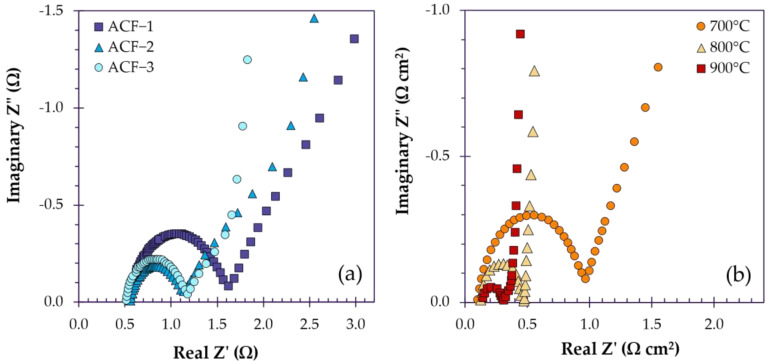
(**a**) EIS testing of ACFs with varying pore distributions, redrawn based on data reported by Abedi et al. [107]. (**b**) EIS testing of ACFs with varying mesoporosities developed at increasing temperatures, redrawn based on data reported by Kim et al. [96].

Often when electrodes are composed primarily of micropores, the rate capability suffers due to restricted ion transport and sluggish diffusion in and out of micropores (Figure 9a). For instance, Abedi et al. prepared ACFs with a BET SSA of 1091 m^2^/g and microporosity of 77%, which achieved a capacitance of 276 F/g at 40 mA/g in 6 M KOH. Upon application of an increase in current density to 4 A/g, the capacitance fell to 114 F/g, losing 59% of its capacity. However, when mesoporosity was increased, ACFs with a BET SSA of 2290 m^2^/g and microporosity of 69% fell by only 29%, from 311 F/g at 40 mA/g to 222 F/g at 4 A/g, owing to their bimodal pore distribution [107]. In the same study, a salient example of the need to tailor the surface morphology to the intended electrolyte is presented: ACF electrodes were trialed in the ionic liquid electrolyte EMIMBF_4_, in which the capacitance was lower (205 F/g at 40 mA/g), and the rate capabilities were much lower (−43% retention in EMIMBF_4_ versus −29% in KOH). Because the ion sizes of EMIMBF_4_ are larger (ionic diameter of 0.8 nm for EMIM^+^ and 0.38 nm for BF_4_^−^ [107]) compared to KOH (ionic diameter of ~0.26 nm [165] for K^+^ and ~0.35 nm [165] for OH^−^), ions likely struggled to access micropores and encountered significant ionic diffusion resistance, and were also hindered by the high viscosity of the electrolyte. During electrochemical impedance spectroscopy (EIS) testing, the test cell failed to enter the Warburg region even at very low frequencies, suggesting that the process is strongly diffusion-limited [107].

**Figure 10:**
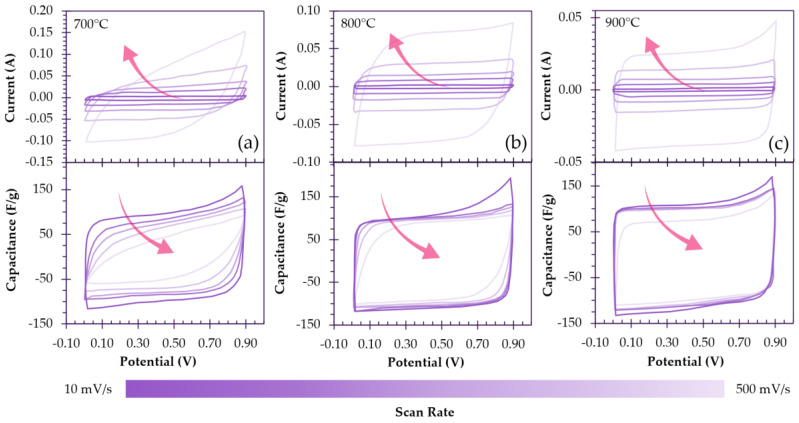
CV curves recorded at various scan rates for ACFs activated at (**a**) 700 °C, (**b**) 800 °C and (**c**) 900 °C. Arrows indicate CVs at increasing scan rates (from 10 mV/s to 500 mV/s), redrawn based on data reported by Kim et al. [96].

As explored in the previous section, when the activation temperature is increased, the development of mesoporosity is favored, which can reduce diffusive resistance. Kim et al. present a clear case in which ACNFs attain increasingly ideal EDLC behavior as mesoporosity is developed and ion transport and diffusion are no longer rate-limiting (Figure 9b). Upon activation of ACNFs from 700 to 900 °C in steam, the mesoporosity increases from 21% to 51%, accompanied by an increasing BET SSA of 723 m^2^/g to 1725 m^2^/g, respectively. ACNFs activated at 700 °C exhibit quasi-rectangular cyclic voltammetry (CV) curves that become increasingly distorted at higher scan rates, demonstrating that (i) charge storage is facilitated almost solely by the electric double layer with minimal pseudocapacitance contribution, and (ii) there is some resistance present upon charge/discharge, suggesting that ion mobility could be hindered in micropores. As the activation temperature is increased to 900 °C and the mesoporosity increases, the degree of rectangularity increases, approaching that of an ideal EDLC, indicating that ionic transport is no longer limiting (Figure 10a–c). Moreover, the rate capability improves from −35% to −15% at elevated scan rates and the ACNFs transition towards purely capacitive behavior, suggesting that diffusive impedance is minimized due to mesopore introduction [96].

### 5.2. Activation Influence on Capacitive and Pseudocapacitive Behavior

**Figure 11:**
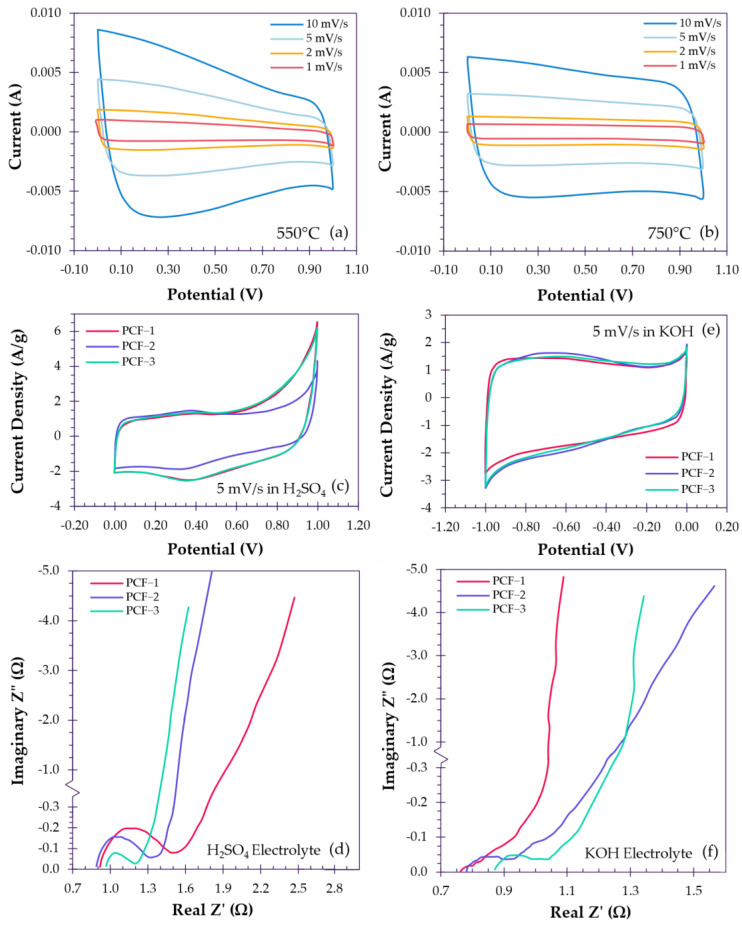
(**a**) CV curves of ACFs with higher heteroatom content and (**b**) lower heteroatom content, redrawn based on data reported by Zhang et al. [112]. (**c**,**d**) CV curves and EIS testing illustrating surface-controlled and (**e**,**f**) diffusion-controlled behavior and kinetics, redrawn based on data reported by Ni et al. [108].

Similarly, in addition to the optimization of the micro- and mesopore composition, higher activation temperatures can also encourage the pursuit of more ideal EDLC behavior through the liberation of heteroatoms from the carbon matrix [114]. For example, Zhang et al. compared the performance of ACFs activated with KOH at 550 °C and 750 °C, where increasing temperature saw a decrease in oxygen-containing functional groups and higher degrees of rectangularity in CV curves. When sweeping ACFs at higher scan rates, CV curves of lower temperature samples exhibited increasing distortion, which was ascribed to the weaker reversibility of pseudocapacitance that arises with the presence of functional groups (Figure 11a,b). Rate capability was also affected when subjected to increasing current densities with 550 °C and 750 °C ACFs achieving 79% retention from 427 F/g to 338 F/g and 91% retention from 224 F/g to 205 F/g from 0.1 A/g to 1 A/g in 6 M KOH, respectively [112]. Functional groups may also be removed by post-activation processing such as hydrogenation, in which ACFs are subjected to reaction with hydrogen at high temperatures, as carried out by Kim et al. [106]. During the reaction, porosity is observed not to be affected, but the removal of oxygen atoms and oxygen-containing functional groups is achieved, resulting in a reduction in pseudocapacitance contribution and enhanced electrochemical properties [106]. However, while ideal EDLC behavior fosters highly favorable kinetics, the energy storage capacity is unfortunately dwarfed by that which can be offered by pseudocapacitance. For instance, Torchała et al. activated carbon fibers using CO_2_ and NH_3_, where CO_2_-activated fibers achieved a BET SSA of 1454 m^2^/g, with lower degrees of oxygen and nitrogen functionalization, while NH_3_-activated fibers achieved a BET SSA of 891 m^2^/g, with higher degrees of oxygen and nitrogen functionalization. Subsequent electrochemical testing revealed that both types of ACF achieved remarkably similar capacitances in two different electrolytes, with CO_2_-ACFs and NH_3_-ACFs achieving 142 F/g and 146 F/g, respectively, in 6 M KOH, and 96 F/g and 91 F/g, respectively, in 0.5 M K_2_SO_4_ [100]. Despite the lower SSA, functionalization with resultant pseudocapacitance is able to compensate for the loss in electric double-layer capacitance [100,115].

In a similar fashion to the enhancement of electric double-layer capacitance, the pairing of a suitable electrolyte with the appropriate surface properties (or vice versa) can promote more favorable interactions [118] that contribute to pseudocapacitance. For example, Ni et al. performed post-activation treatments to yield ammoniated and nitrogen-doped functionalized ACFs, which were evaluated in 6 M KOH and 1 M H_2_SO_4_ along with unmodified ACFs. Based on kinetic analyses, ACFs in 6 M KOH appeared to be primarily surface controlled, which is corroborated by the quasi-rectangular CV curves, yielding capacitances of ~301 F/g for all three samples. However, when tested in 1 M H_2_SO_4_, kinetic analyses revealed a shift to a dominance in diffusion-controlled capacitance, which is corroborated by a pronounced distortion in the CV curves (Figure 11c,d). In replacing KOH with H_2_SO_4_, capacitance was measured to be 482 F/g, 289 F/g and 467 F/g for unmodified, ammoniated and nitrogen-doped ACFs, respectively [108]. The significant jump in capacitance for unmodified and nitrogen-doped ACFs (both containing similar oxygen and nitrogen contents) is explained by the introduction of redox-conducive protons that greatly enhance pseudocapacitive interactions with nitrogen- and oxygen-containing functional groups, which were lacking in the basic solution, though the increase in charge transfer resistance is evident from EIS tests (Figure 11e,f). Despite having the highest nitrogen and oxygen content, the capacitance obtained by ammoniated ACFs experienced a decline, the cause of which was neglected in the discussion [108].

## 6. Summary and Perspectives

To summarize, we have explored methods for introducing porosity onto pitch-based carbon fibers and carbon nanofibers, necessitated by the limited reviews on pitch-derived ACFs. We briefly introduced the foundational theories of the electric double layer to understand the necessity for porous structures in enhancing capacitance and energy storage efficiency and evaluated the performance of pitch-based activated carbon fibers in supercapacitor applications.

Our evaluation revealed that activation, both physical (e.g., steam or CO_2_) and chemical (e.g., KOH), remains the predominant method for pore generation on pitch-based carbon fibers. Key factors affecting activation include fiber diameter, where CNFs exhibit superior activation rates due to their inherently higher surface area compared to conventional CFs. Often elevated in melt-spun fibers, crystallinity poses a challenge by reducing defect sites and discontinuities and impeding activation agent penetration, although chemical activation via intercalation can disrupt ordered structures to facilitate porosity. Due to the endothermic nature of activation reactions (particularly physical activation), mesopore formation is promoted at elevated temperatures through more aggressive etching, while prolonged activation times enhance carbon removal but result in diminished yields. Additionally, the incorporation of inorganic nanoparticles can catalyze pore expansion, particularly in physical activation, leading to mesoporous networks and even rut-like channels that enhance ion transport and access. Furthermore, alternative methods to obtain porosity include templating strategies, as well as exploitation of the intrinsic porosity that can develop during the fiber formation process.

Performance-wise, microporous structures in pitch-based ACFs and ACNFs afford the confinement that is essential for elevated capacitances. However, excessive microporosity compromises rate capability by restricting ion diffusion and mobility, resulting in suboptimal power delivery. Hierarchical pore distributions, which incorporate meso- and macropores, mitigate these limitations by providing efficient transport pathways, yielding faster charge/discharge kinetics and more ideal EDLC behavior. Activation can also alter the electrochemical behavior, either reinforcing EDL dominance through surface group removal or introducing pseudocapacitive contributions via oxygen- or nitrogen-containing functionalities.

In performing this review, the scarcity of pore-introduction methods, beyond activation, specifically for pitch-based carbon fibers, was apparent, which thereby invites future studies to explore alternative avenues such as templating to diversify the engineering of porosity for this material. Moreover, differences in reported supercapacitor metrics—stemming from varied scan rates, current densities, mass loadings, electrode design and capacitance calculation bases (e.g., per electrode vs. active material)—hamper direct comparisons and benchmarking. It is clear that the establishment and utilization of standardized reporting protocols would be invaluable for future studies to encourage streamlined and accurate comparison between studies.

## Figures and Tables

**Table 1:** Properties and characteristics of various pitch-based activated carbon fibers and their activation conditions.

Activation Method	Precursor Material	Activation Agent ^†^	Temperature (°C)	Time (h)	BET Surface Area (m^2^/g)	Microporosity (%)	Yield (%)	Ref.
Physical Activation	Pitch	H_2_O	900	0.67	3230	43.9%	6%	[89]
	Pitch	H_2_O	900	6.00	2630	63.4%	15%	[90]
	Isotropic Petroleum Pitch	H_2_O	700	1.00	2222	99.7%	–	[91]
	Isotropic Pitch	CO_2_ + H_2_O	–	3.00	2129	64.4%	7%	[92]
	Isotropic Pitch (from PFO)	H_2_O	900	1.00	2053	51.3%	–	[93]
	Pitch/PAN	H_2_O	900	1.00	1877	49.2%	–	[94]
	Isotropic Pitch	CO_2_	900	–	1843	–	20%	[95]
	Pitch	H_2_O	800	1.00	1747	59.8%		
	Pitch/PAN	H_2_O	900	1.00	1724	49.2%	–	[96]
	Petroleum Pitch	H_2_O	1075	–	1123	–	53%	[97]
	Pitch/PAN	H_2_O	800	–	936	55.0%	–	[98]
	Pitch	H_2_O	900	1.00	920	92.6%	–	[99]
	Anthracene Oil-Based Isotropic Pitch	NH_3_	850	0.50	891	93.0%	50%	[100]
	Petroleum Pitch/PAN Nickel Doped	CO_2_	900	1.00	750	50.1%	–	[101]
	Isotropic/Mesophase Pitch	CO_2_	840	6.00	–	–	–	[102]
Chemical Activation	Pitch/PVP	KOH	900	2.00	3311	–	–	[103]
	Isotropic Pitch	KOH	950	1.00	2672	57.2%	42%	[104]
	Pitch	NaOH	900	1.00	2460	63.4%	17%	[105]
	Mesophase Pitch	KOH	700	2.00	2353	–	–	[106]
	Asphaltene	KOH	800	2.00	2290	69.0%	–	[107]
	Asphaltene	KOH	800	1.00	2233	84.9%	–	[108]
	Low SP Pitch and Polystyrene	KOH	900	3.00	2169	–	12%	[109]
	Isotropic Pitch	KOH	700	1.00	1770	83.1%	49%	[110]
	Petroleum Pitch/MWCNTs	KOH	750	3.00	1665	53.0%	–	[28]
	Low SP Pitch	KOH	650	1.00	1504	75.7%	78%	[111]
	Mesophase Pitch	KOH	550	1.00	1222	83.7%	74%	[112]
	Pitch	KOH	750	3.00	1148	89.1%	–	[113]
	Pitch	KOH	800	3.00	987	84.3%	–	[114]
	Coal Tar Pitch	K_3_C_6_H_5_O_7_	700	–	550	–	–	[115]
	Isotropic Pitch	KOH	700	2.00	286	98.0%	–	[116]
Thermal Treatment	PAN/Pitch	–	1000	–	966	–	–	[83]
	Pitch/PAN Boron and Mn Doped	–	800	1.00	718	–	–	[117]
	Isotropic Petroleum Pitch/PAN Boron Doped	–	800	1.00	641	43.0%	–	[118]
	Pitch/PAN Mn Doped	–	800	1.00	620	–	–	[119]
Not Specified	Pitch	–	–	–	1640	88.5%	–	[120]

^†^ H_2_O = steam, CO_2_ = carbon dioxide, NH_3_ = ammonia, KOH = potassium hydroxide, NaOH = sodium hydroxide, K_3_C_6_H_5_O_7_ = potassium citrate.

## Data Availability

No new data were created or analyzed in this study.

## Abbreviations

The following abbreviations are used in this manuscript:

ACF	Activated Carbon Fiber
ACNF	Activated Carbon Nanofiber
CF	Carbon Fiber
CV	Cyclic Voltammetry
ECAS	Electrochemically Accessible Surface Area
EDL	Electric Double Layer
EDLC	Electric Double-Layer Capacitor
EIS	Electrochemical Impedance Spectroscopy
ESD	Energy Storage Device
IHP	Inner Helmholtz Plane
MW	Molecular Weight
OHP	Outer Helmholtz Plane
PAN	Polyacrylonitrile
